# ShinyBioHEAT: an interactive shiny app to identify phenotype driver genes in *E.coli* and *B.subtilis*

**DOI:** 10.1093/bioinformatics/btad467

**Published:** 2023-07-31

**Authors:** Chen Wang, Harikumar Govindarajan, Panagiotis Katsonis, Olivier Lichtarge

**Affiliations:** Department of Molecular and Human Genetics, Baylor College of Medicine, Houston, TX 77030, United States; Department of Molecular and Human Genetics, Baylor College of Medicine, Houston, TX 77030, United States; Department of Molecular and Human Genetics, Baylor College of Medicine, Houston, TX 77030, United States; Department of Molecular and Human Genetics, Baylor College of Medicine, Houston, TX 77030, United States; Quantitative and Computational Biosciences Graduate Program, Baylor College of Medicine, Houston, TX 77030, United States; Verna and Marrs McLean Department of Biochemistry and Molecular Biology, Baylor College of Medicine, Houston, TX 77030, United States; Cancer and Cell Biology Graduate Program, Baylor College of Medicine, Houston, TX 77030, United States; Computational and Integrative Biomedical Research Center, Baylor College of Medicine, Houston, TX 77030, United States

## Abstract

**Summary:**

In any population under selective pressure, a central challenge is to distinguish the genes that drive adaptation from others which, subject to population variation, harbor many neutral mutations *de novo*. We recently showed that such genes could be identified by supplementing information on mutational frequency with an evolutionary analysis of the likely functional impact of coding variants. This approach improved the discovery of driver genes in both lab-evolved and environmental *Escherichia coli* strains. To facilitate general adoption, we now developed ShinyBioHEAT, an R Shiny web-based application that enables identification of phenotype driving gene in two commonly used model bacteria, *E.coli* and *Bacillus subtilis*, with no specific computational skill requirements. ShinyBioHEAT not only supports transparent and interactive analysis of lab evolution data in *E.coli* and *B.subtilis*, but it also creates dynamic visualizations of mutational impact on protein structures, which add orthogonal checks on predicted drivers.

**Availability and implementation:**

Code for ShinyBioHEAT is available at https://github.com/LichtargeLab/ShinyBioHEAT. The Shiny application is additionally hosted at http://bioheat.lichtargelab.org/.

## 1 Introduction

*Escherichia coli* and *Bacillus subtilis* are ideal model organisms for genotype–phenotype studies due to their unique advantages. They grow fast and benefit from a vast array of genetic editing techniques (Swings *et al.* 2018, Choudhury *et al.* 2020, Csörgő *et al.* 2020, Zhang *et al.* 2020) and bioinformatics databases (Keseler *et al.* 2021, Szklarczyk *et al.* 2019, Tierrafría *et al.* 2022). Increasingly, studies that seek to pinpoint the driver genes of phenotypes of interest combine adaptive laboratory experiments (ALEs) with next-generation sequencing (Tenaillon *et al.* 2016, Zeigler and Nicholson 2017, van den Bergh *et al.* 2018, Bruckbauer *et al.* 2019, Karve and Wagner 2022). Typically, these studies rank genes based on their relative mutational frequency in parallel streams of replications. This sole use of mutational frequency ignores additional information on the functional impact of coding variants, however, and reduces the power to detect secondary diver genes.

To improve the identification of driver genes, we recently developed a new EA integration approach (Marciano *et al.* 2022), which exploits the Evolutionary Action (EA) score (Katsonis and Lichtarge 2014) for the likely impact of any missense mutation in any given protein from past evolutionary history. EA scores tend to correlate well with experimental mutagenesis studies in objective, blinded challenges evaluated by third parties (Katsonis and Lichtarge 2019) and to predict the harmful effect of mutations in diverse applications (Katsonis *et al.* 2022). In a direct test of its potential for elucidating ALE-induced phenotypes in *E.coli*, EA integration improved phenotype driver gene discovery compared with frequency-based method, especially so in the clinical/environmental datasets (Marciano *et al.* 2022).

To broaden access to our method, we developed a user-friendly R Shiny (Chang *et al.* 2022) package, ShinyBioHEAT (Biodetection of High Evolutionary Action Targets), using golem framework (Fay *et al.* 2022) which allows easy installation across platforms and running locally. The main feature for ShinyBioHEAT is to identify phenotype driving genes in *E.coli* and *B.subtilis* from sequencing data by combining EA scores with frequency statistics (Marciano *et al.* 2022). Additional modules are developed to allow sequential analysis through STRING for the top predicted genes and visualization of mutational profiles on protein structures.

## 2 Features

### 2.1 Driver gene analysis module

**Figure 1.**
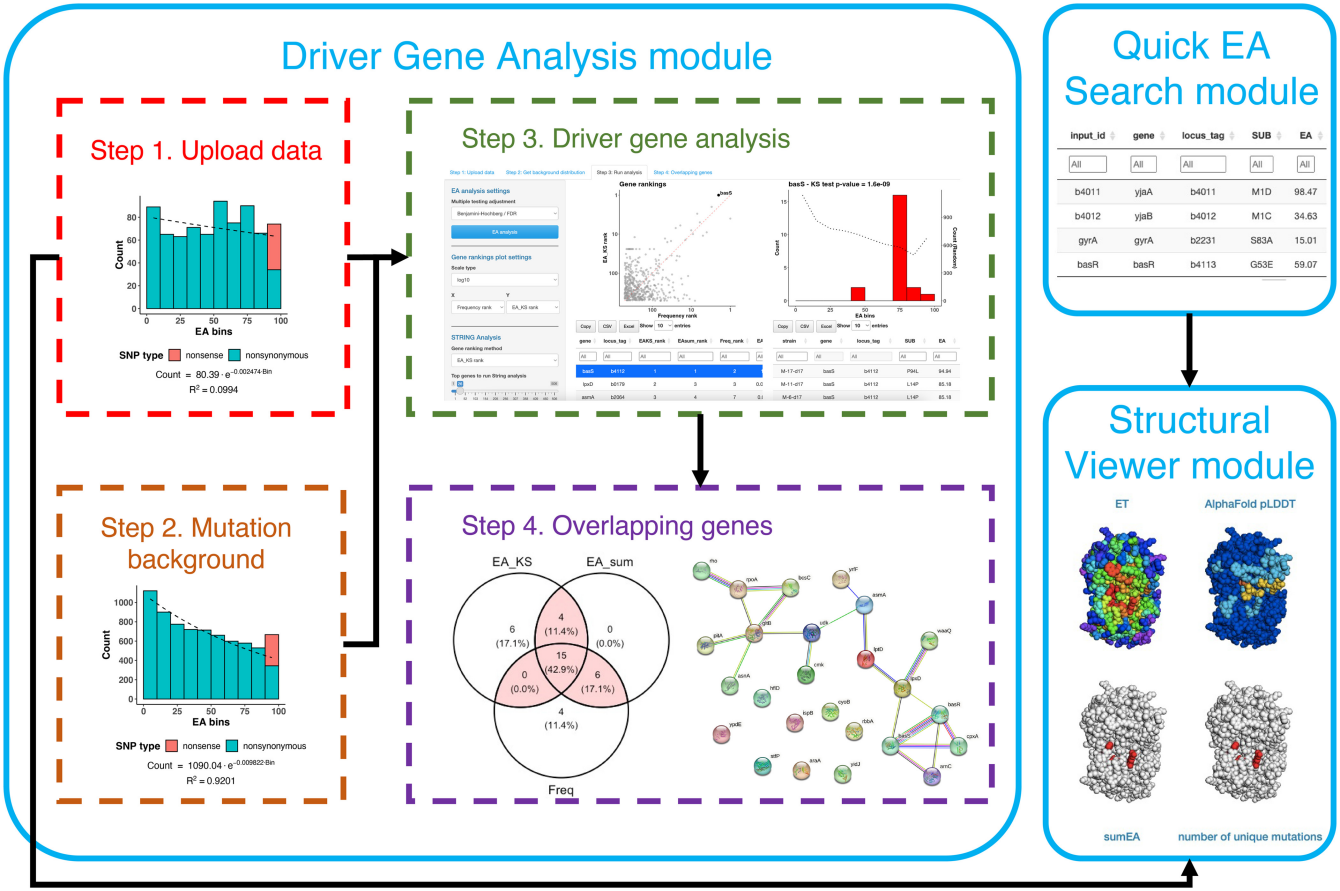
Graphical overview of the functional modules in ShinyBioHEAT. Data flow is indicated with black arrows.

This is the main module of ShinyBioHEAT application (Fig. 1), which allows the identification of driver genes from *E.coli* and *B.subtilis* sequencing data using EA integration and a frequency-based method. It currently supports three reference genomes: *E.coli* MG1655 (RefSeq: NC_000913.3), *E.coli* REL606 (RefSeq: NC_012967.1), and *B.subtilis* 168 (RefSeq: NC_000964.3). Sequencing data can be uploaded as variant call format (VCF), amino acid substitutions, or breseq GenomeDiff (GD) format (Deatherage and Barrick 2014). The amino acid substitutions will be determined if VCF format is used. EA scores are then assigned to each missense mutation, which will be compared with a mutation background to identify the driver genes. Mutation background can be generated through randomly simulated mutations in the selected reference genome or a custom set of mutations.

To account for functional impact of mutations in driver gene prediction, EA integration was implemented with two different approaches: EA_KS and EA_sum. They compare the EA distribution of mutations for each gene in the evolve strains against the mutation background, and then prioritize genes that accumulate more impactful mutations during the adaptation. As an orthogonal control, a frequency-based method is also implemented, which ranks the genes based on mutation count and gene length.

To further evaluate the top-ranked genes and narrow down the genes for experimental validation, an interactive Venn diagram is implemented to allow identification of overlapping predictions by the three approaches. Genes that are highly ranked by different methods are more likely driver genes. In addition, driver genes tend to cluster well in protein–protein interaction networks. We utilize the STRING API (Szklarczyk *et al.* 2019) to allow quick STRING PPI enrichment test on the top or overlapping predictions.

### 2.2 Quick EA search

The Quick EA search module allows user to identify the EA scores for missense mutations in the selected reference genome on-the-fly. EA consistently predicts well the protein mutational impact in objective challenges (Katsonis and Lichtarge 2019), which makes it a useful resource.

### 2.3 Structure viewer

Visualizing mutations on the protein structure provides valuable insights on the molecular mechanism of protein function and can guide mutagenesis studies. The recent advances in protein structure predictions give access to high-quality protein structures for nearly all *E.coli* and *B.subtilis* proteins (Jumper *et al.* 2021). The structure viewer displays the AlphaFold protein structures using r3dmol library (Rego and Koes 2015, Su and Johnston 2022) with four different coloring schemes: Evolutionary Trace (ET), pLDDT, sumEA, and number of unique mutations. ET estimates the importance of a residue position in a protein by examining its evolutionary history (Lichtarge *et al.* 1996). Clustering of important ET residues is a hallmark for protein functional site (Wilkins *et al.* 2013; Wang *et al.* 2021). PLDDT is the structure prediction accuracy score from AlphaFold. SumEA and number of unique mutations project the evolutionary burden in the evolved strains. A Pymol session file with the same coloring scheme is also generated to allow closer examination on Pymol (Schrödinger LLC 2015).

An example study case using ShinyBioHEAT is provided in the Supplementary Data.

## 3 Conclusion

ShinyBioHEAT is a user-friendly Shiny interface to identify phenotype driver genes in adapted *E.coli* with minimal coding experience. It also provides downstream analyses through STRING database and color mapping to AlphaFold protein structures. It is freely distributed as an R package under the MIT license at https://github.com/LichtargeLab/ShinyBioHEAT.

## Supplementary Material

btad467_Supplementary_Data

## Data Availability

The data underlying this article are available in its online supplementary material and its GitHub repository.
