# Supplementary material for: ShinyBioHEAT: an interactive shiny app to identify phenotype driver genes in E.coli and B.subtilis
Source: Bioinformatics. 2023 Jul 31;39(8):btad467. doi: 10.1093/bioinformatics/btad467 (PMC10412404; doi:10.1093/bioinformatics/btad467)
Supplement: btad467_Supplementary_Data [file btad467_supplementary_data.docx]

**A case study using ShinyBioHEAT**

1. How to start

A live version of ShinyBioHEAT is hosted at <http://bioheat.lichtargelab.org/>.

R version >= 4.0.0 is required to install a local copy of ShinyBioHEAT.

To install and run ShinyBioHEAT locally use the following commands:

*if (! "devtools" %in% installed.packages()) {*

*install.packages(devtools)*

*}*

*devtools::install_github("LichtargeLab/ShinyBioHEAT")*

*library(ShinyBioHEAT)*

*run_app()*

A new webpage will pop-up in the browser followed by a pop-up window, where users are allowed to select the reference genome (**Fig. S1a**). Three reference genomes are currently supported including *E. coli* MG1655 (RefSeq: NC_000913.3), *E. coli* REL606 (RefSeq: NC_012967.1) and *B. subtili* 168 (RefSeq: NC_000964.3). After the application loads the required data (usually takes several seconds) the Background module of ShinyBioHEAT (**Fig. S1b**) is then displayed.


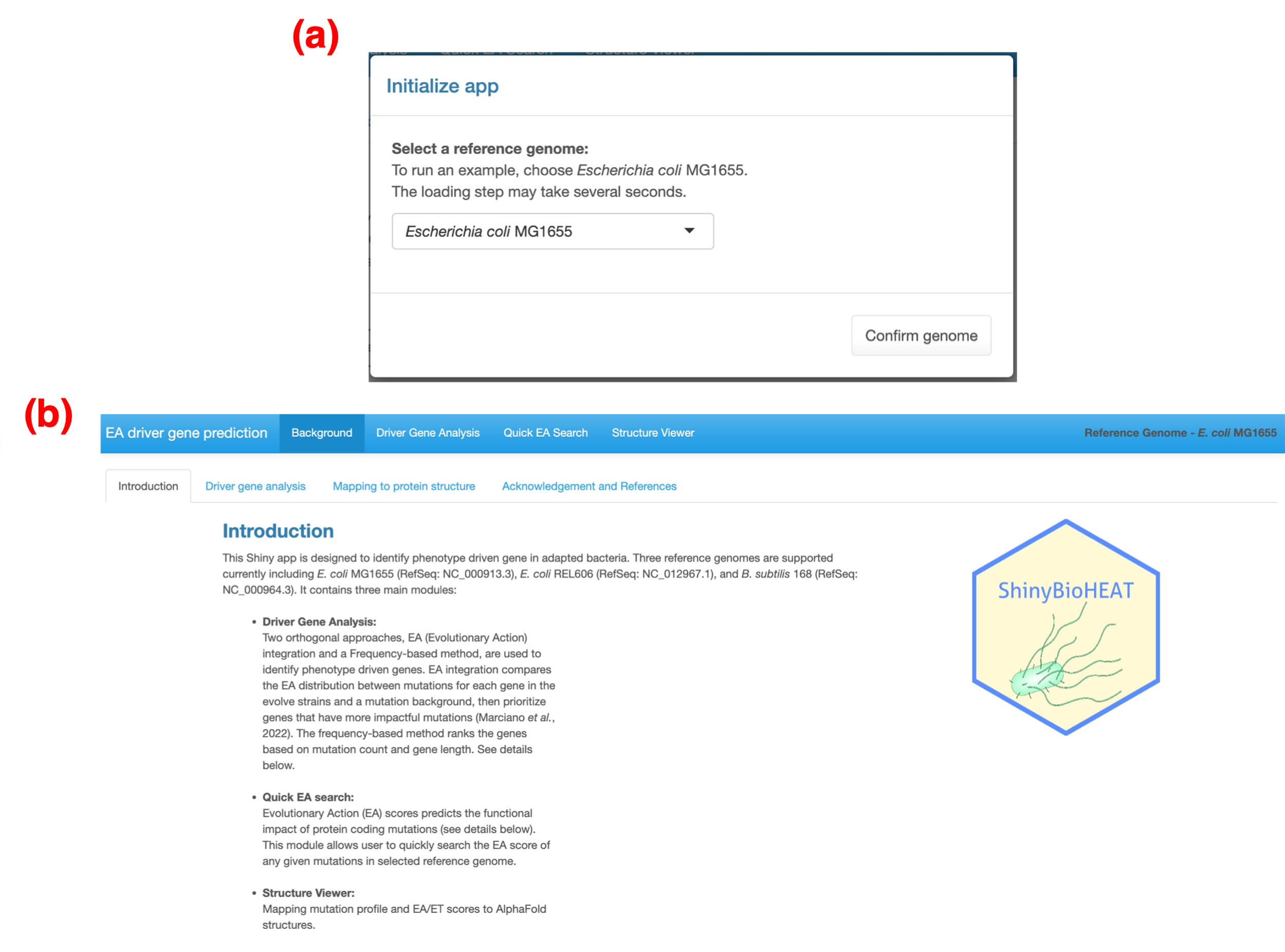


**Figure S1.** The Home page of ShinyBioHEAT.

1. Prepare input data

The ShinyBioHEAT takes three types of mutation data: variant call format (VCF), an amnio acid substitution format (SUB), and the GenomeDiff (GD) format from breseq (**Fig. S2**).

**Figure S2.** Examples of the VCF and SUB input formats.

VCF files contain 6 columns that are separated by tab: “#CHROM”, “POS”, “ID”, “REF”, “ALT”, “QUAL”, “FILTER”, and “INFO”. SUB files contain 2 mandatory columns that are comma separated (CSV): “input_id” and “SUB”. Both locus tag and gene name can be used as “input_id”, but we recommend using locus tag for less ambiguity. “SUB” column stores the amino acid substitution (e.g. C84R). Please refer to the breseq (Deatherage & Barrick, 2014) website (https://barricklab.org/twiki/pub/Lab/ToolsBacterialGenomeResequencing/documentation/gd_format.html) for details on GD format.

1. Running ShinyBioHEAT

As a case study, we will try to identify colistin resistance driver genes from the sequencing information of 20 independently evolved colistin resistant *E. coli* strains (Marciano et al., 2022).Those strains were adapted in increasing concentration of colistin in the presence of nucleotide analogs (2-aminopurine and zebularine) to increase mutation rate (Ang et al., 2016).

**Step 1. Upload data**

In the “Driver Gene Analysis” module, we will use VCF format as input (**Fig. S3a**). The sequencing information can be downloaded through the “Download example VCF files” link (**Fig. S3b**). Extract the zip file, the upload all the VCF files to the application (**Fig. S3c**). Multiple files can be selected by holding the ctrl/command key. After submitting the files, mutations in the VCF files will be annotated. Missense mutations will be assigned with Evolutionary Action (EA) scores (Katsonis & Lichtarge, 2014). EA scores range from 0-100. Mutations with higher EA scores are predicted to have more functional impact. Non-sense mutations are arbitrarily assigned with EA = 100.


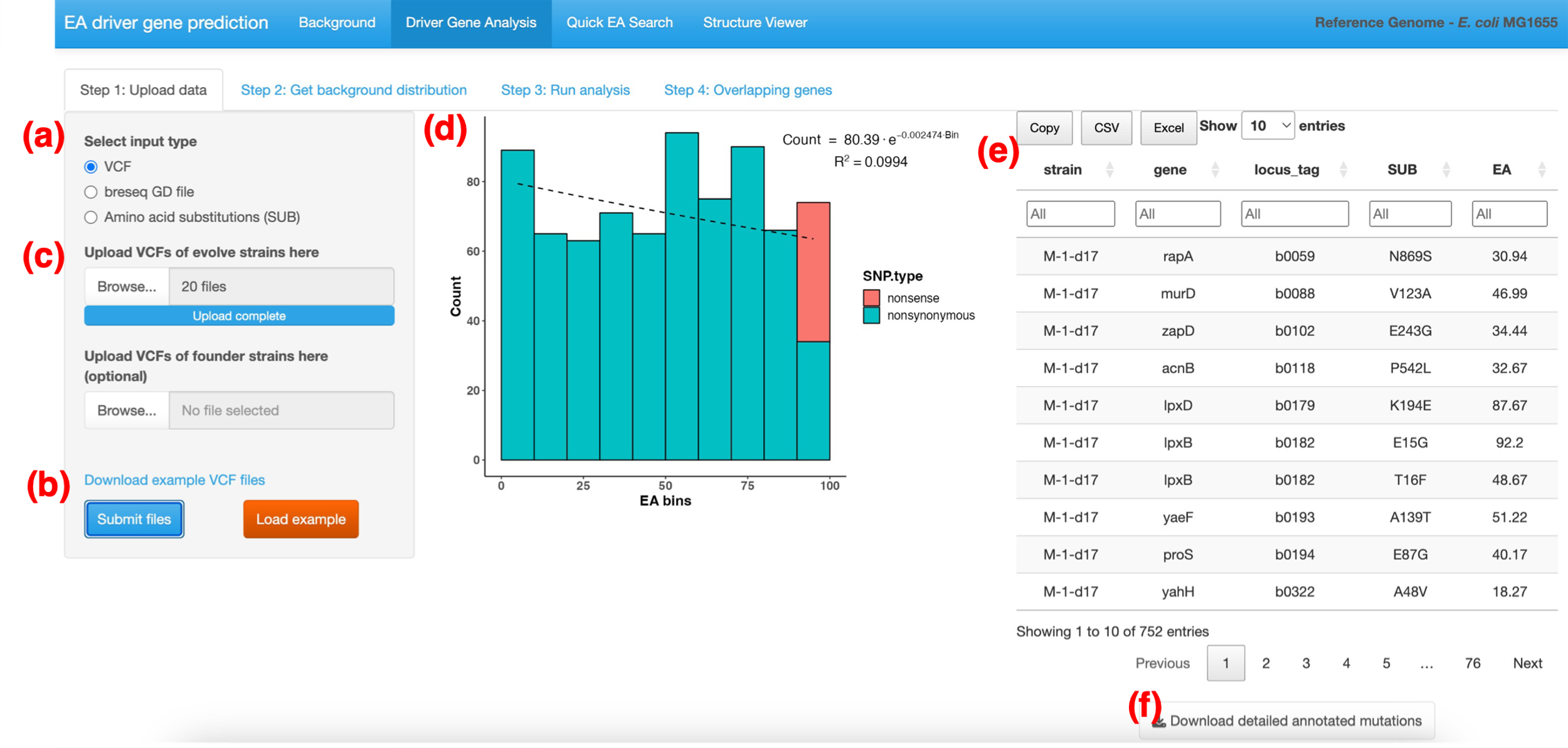


**Figure S3.** Interface for uploading sequencing data. (a) Options to select input file type. (b) Link to download example VCF file. (c) File upload. (d) The EA distribution of all mutations in the evolve strains after removing mutations in the founder. (e) All coding mutations in the evolved strains. (f) Download detailed mutations

The distribution of the EA scores in all coding mutations (**Fig. S3d**) are graphed. Mutations obtained without selection have an exponential decay pattern in their EA distribution (see Step 2 for detail). As expected, the mutations observed in Fig. S3d have their EA distribution shifted toward higher values, suggesting these *E. coli* strains are under positive selection. All coding mutations are listed in an interactive table (**Fig. S3e**). The detailed annotated mutations, including non-coding mutations and silent mutations, can be accessed through the “Download detailed annotated mutations” button (**Fig. S3f**).

**Step 2. Generate background distribution**

A mutation background is required for EA integration analysis. It can be generated by randomly simulating mutations in the *E. coli* genome or through custom mutation files. Users can specify the number of random mutations in each strain and the number of simulated strains (**Fig. S4a**). It is recommended to use at least 1000 mutations in the mutation background. The “Advanced settings” allow users to customize the percent of transition mutations, and whether to exclude non-coding regions from the simulation (**Fig. S4b**). If all other settings are fixed, using the same “random seed” values will produce the same set of random mutations.

Here we simulated 10 *E. coli* MG1655 strains, each with 1000 coding mutations (**Fig. S4**). As shown in Fig. S4c, the EA distribution of randomly simulated nonsynonymous mutations has an exponential decay pattern, which is different from the EA distribution of mutations in the evolve strains (**Fig. S3d**).


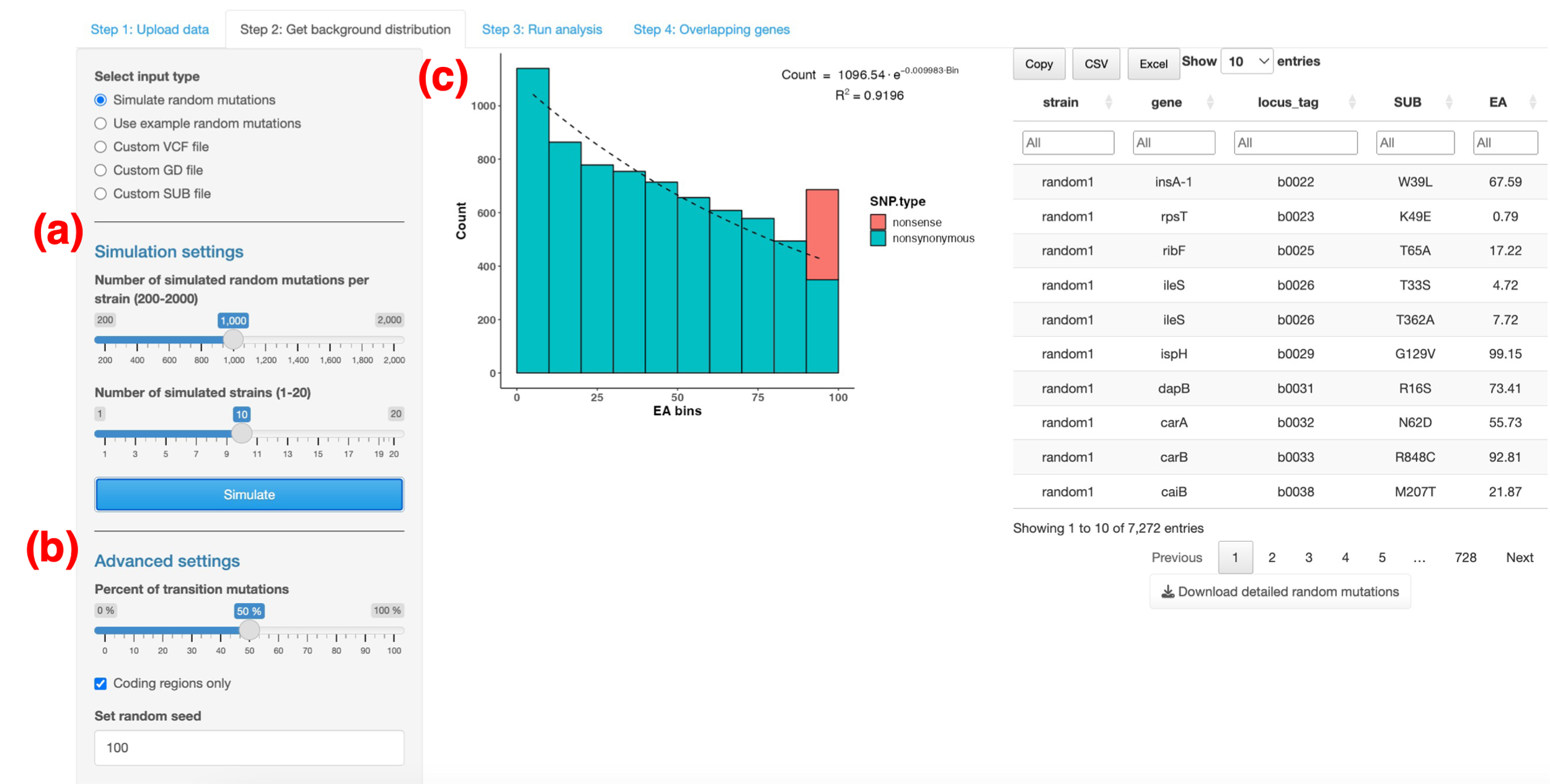


**Figure S4.** The interface for simulating random mutation background. (a) Options to control simulated mutation count. (b) Options to control Ti/Tv ratio, mutation regions and random seed. (c) The EA distribution of simulated random mutations.

**Step 3. Run analysis**

Two EA integration approaches (EA_KS and EA_sum) and a frequency-based approach are implemented to detect driver genes in the evolved *E. coli* strains (Marciano et al., 2022). After submitting the input files and simulating the background distribution, navigate to the “Step 3. Run analysis” tab, select the desired multiple testing adjustment method (**Fig. S5a**), then click on the “EA analysis” button. Note that the p values from KS test are sensitive to number of mutations used in the random background.

When the analysis completes, the gene rankings will be plotted (**Fig. S5b**). Options on the left side allows user to control the which methods are used in the axes and the scale (**Fig. S5c**). The detailed gene rankings are listed in the table below the scatter plot (**Fig. S5d**). The table and the scatter plot are interactively connected. Clicking a dot (gene) on the scatter plot will highlight that gene on the plot and in the table and vice versa. In addition, the EA distribution of the highlighted gene is shown in the upper-right plot (**Fig. S5e**) and is compared against the mutation background (dotted line). All the mutations occurred in the highlighted gene are listed in the lower-right table (**Fig. S5f**). In the colistin resistance example dataset, waaQ is highly ranked by EA integration and the frequency-based methods. Its EA distribution is shifted towards high values comparing to the background. These suggest that waaQ might be a driver gene that contribute to colistin resistance in *E. coli*.

Genes contributing to the same phenotype often involve in the same functional pathways. If the top ranked genes truly contribute to the phenotype of interest, they should cluster more significantly in a protein-protein interaction network. The “STRING Analysis” feature allows user to query the top predicted genes from each method in STRING database (Szklarczyk et al., 2019) (**Fig. 5g**).


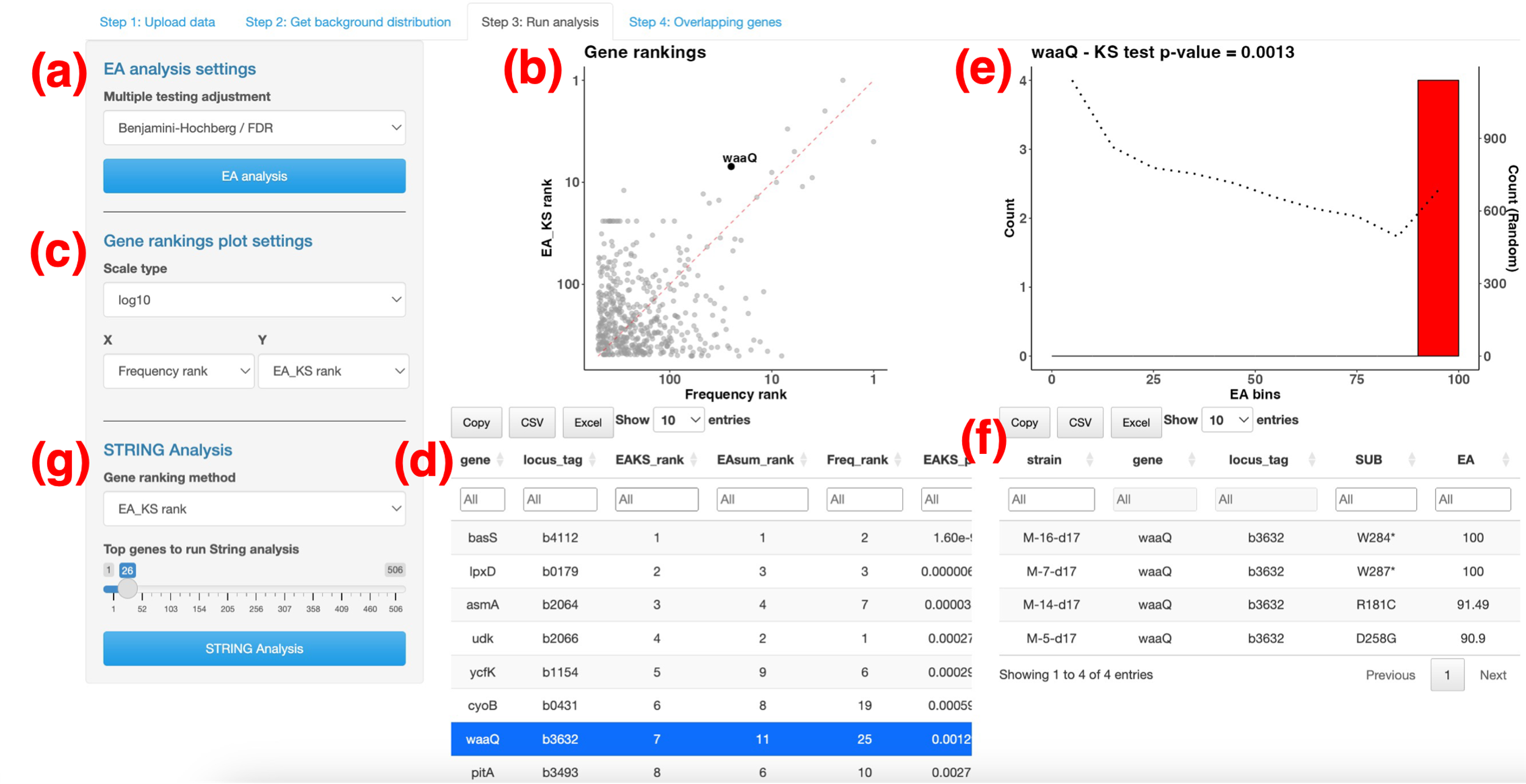


**Figure S5.** Graphical user interface for driver gene analysis. (a) Multiple testing adjustment method (b) An interactive plot showing the gene rankings by different methods. (c) Options to control Gene rankings plot. (d) Gene ranking table showing the predictions from each method and related statistics. This table is dynamically linked with the gene rankings scatter plot. (e) EA distribution of mutations in the highlighted gene. Mutation background showing as dot. (f) Mutations observed in the highlighted gene. (g) STRING analysis on the top ranked genes.

**Step 4. Overlapping genes**

We can check the overlapping genes from the top predictions by all three methods using a Venn diagram (**Fig. S6a**). Genes that are ranked highly by multiple methods are more likely to be true driver gens. Click on a section on the Venn diagram to highlight that region. Genes in the highlighted regions are listed in the right-side table (**Fig. S6b**). The highlighted genes in the Venn diagram can be queued in STRING database (Szklarczyk et al., 2019).

In the colistin resistance example dataset, 15 genes are precited in the top 5% by all three methods. Those genes cluster well in the STRING network with PPI enrichment, with p-value = 0.013. BasS and basR are the two most common driver genes for colistin resistance (Poirel et al., 2017) (**Fig. S6c**, green box). WaaQ (**Fig. S6c**, red box) connects with them at high confident level, further suggesting it might contribute to colistin resistance.


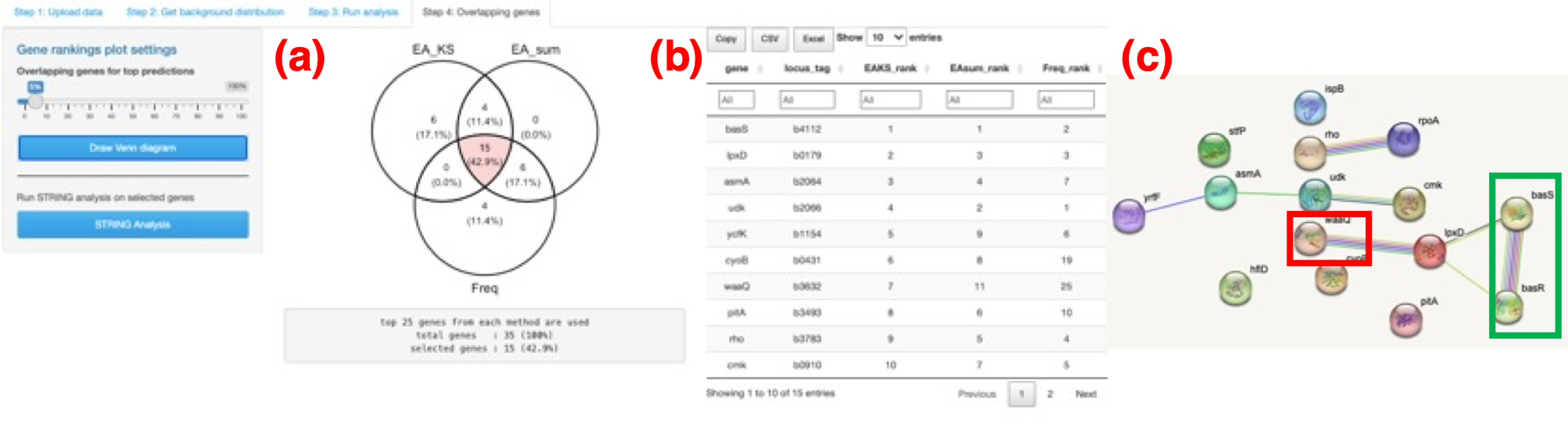


**Figure S6.** Interface for analyzing overlapping genes. (a) Venn diagram showing the overlapping genes. Click on the sections in the Venn diagram to select/deselect. (b) Genes that are highlighted in the Venn diagram. (c) STRING analysis on colistin resistance genes that are precited in the top 5% by all three methods.

**Additional Step: Visualizing mutations on protein structure**

The “Structure Viewer” module allows users to visualize mutations and their EA scores on AlphaFold protein structures (Jumper et al., 2021). The viewer can input mutations from the evolve strains or from the “Quick EA Search” module (**Fig. S7a**). The structures are colored with 4 different color schemes (**Fig. S7b**). The ET coloring highlight functional important positions as warmer (red) colors. Cluster of important ET residues on the protein surface is a proven indicator of protein functional site (Wilkins et al., 2013; Yao et al., 2006). The AlphaFold pLDDT scores indicate the structure prediction accuracy. The sumEA and unique mutation count coloring suggest the evolutionary burden observed in the input data. If a protein has high evolutionary burden (red in sumEA) in the protein functional site (a warm cluster in ET), then the protein function is highly likely to be altered in the evolve strains.

A Pymol session file is also generated at the same time (**Fig. S7c**) to allow the structure mapping to be further evaluated in a more powerful molecular visualization system (Schrödinger, LLC, 2015) (**Fig. S7d**).

Fig. S7b maps 4 waaQ mutations in the evolve strains to the protein structure. ET highlights the potential functional site (dotted circle). 3 of the 4 high EA mutations occurred in this region, suggesting these mutations have a high chance of altering the protein function. This result combines with the consistently high rank by all three method and the connection to known drivers in STRING database, makes waaQ an ideal candidate gene for colistin resistance.


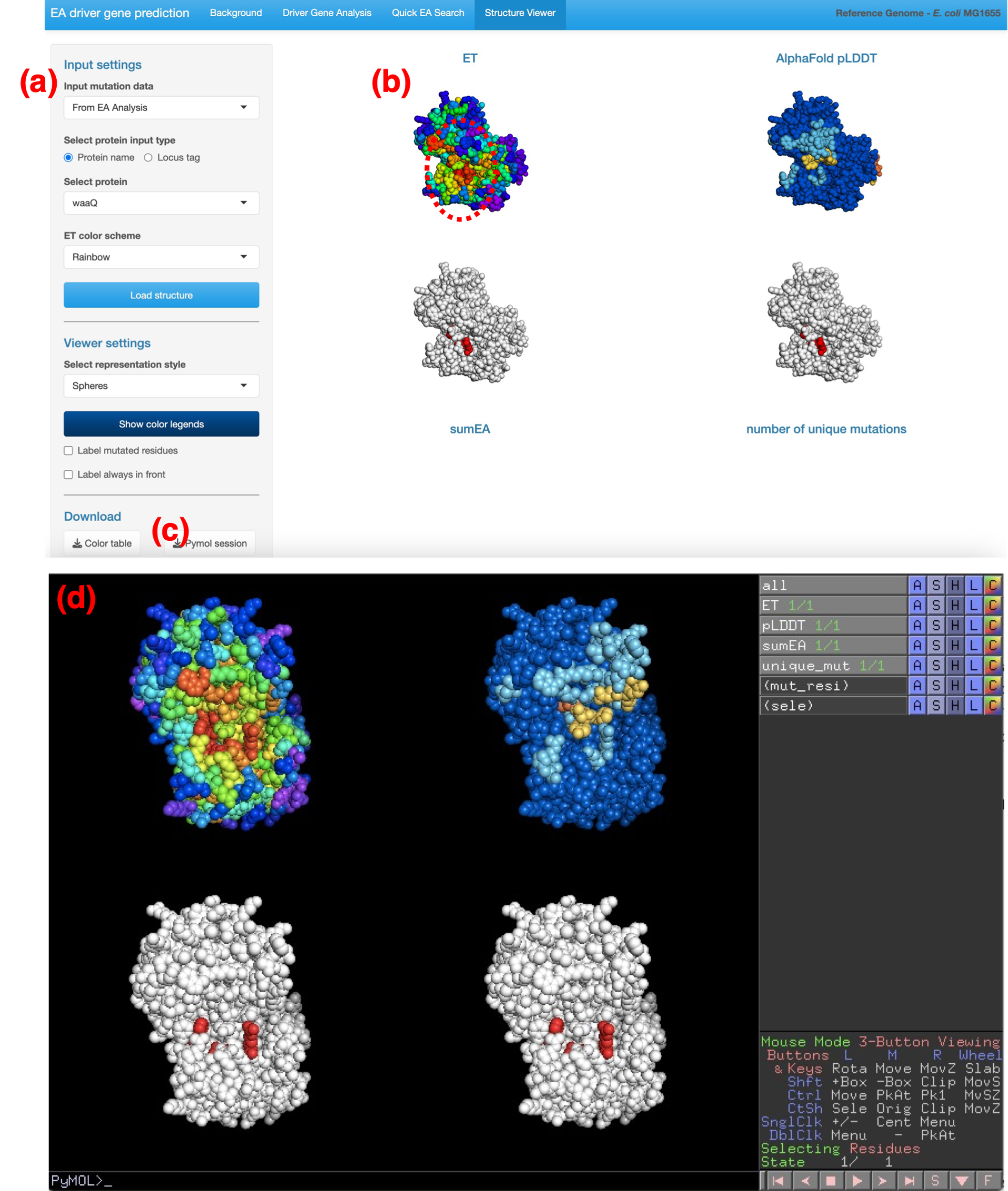


**Figure S7.** Mapping mutation data on to protein structure. (a) Source of mutation data. (b) 4 color mapping of on the protein structure. (c) Download link for Pymol session file. (d) Pymol session that reflects the same coloring scheme.

**References**

Ang, J., Song, L. Y., D’Souza, S., Hong, I. L., Luhar, R., Yung, M., & Miller, J. H. (2016). Mutagen Synergy: Hypermutability Generated by Specific Pairs of Base Analogs. *Journal of Bacteriology*, *198*(20), 2776–2783. https://doi.org/10.1128/JB.00391-16

Deatherage, D. E., & Barrick, J. E. (2014). Identification of Mutations in Laboratory-Evolved Microbes from Next-Generation Sequencing Data Using breseq. In *Methods in Molecular Biology* (pp. 165–188). https://doi.org/10.1007/978-1-4939-0554-6_12

Jumper, J., Evans, R., Pritzel, A., Green, T., Figurnov, M., Ronneberger, O., Tunyasuvunakool, K., Bates, R., Žídek, A., Potapenko, A., Bridgland, A., Meyer, C., Kohl, S. A. A. A., Ballard, A. J., Cowie, A., Romera-Paredes, B., Nikolov, S., Jain, R., Adler, J., … Hassabis, D. (2021). Highly accurate protein structure prediction with AlphaFold. *Nature*, *May*, 1–12. https://doi.org/10.1038/s41586-021-03819-2

Katsonis, P., & Lichtarge, O. (2014). A formal perturbation equation between genotype and phenotype determines the Evolutionary Action of protein-coding variations on fitness. *Genome Research*, *24*(12), 2050–2058. https://doi.org/10.1101/gr.176214.114

Marciano, D. C., Wang, C., Hsu, T.-K., Bourquard, T., Atri, B., Nehring, R. B., Abel, N. S., Bowling, E. A., Chen, T. J., Lurie, P. D., Katsonis, P., Rosenberg, S. M., Herman, C., & Lichtarge, O. (2022). Evolutionary action of mutations reveals antimicrobial resistance genes in Escherichia coli. *Nature Communications*, *13*(1), 3189. https://doi.org/10.1038/s41467-022-30889-1

Poirel, L., Jayol, A., & Nordmann, P. (2017). Polymyxins: Antibacterial Activity, Susceptibility Testing, and Resistance Mechanisms Encoded by Plasmids or Chromosomes. *Clinical Microbiology Reviews*, *30*(2), 557–596. https://doi.org/10.1128/CMR.00064-16

Schrödinger, LLC. (2015). *The {PyMOL} Molecular Graphics System, Version~1.8*.

Szklarczyk, D., Gable, A. L., Lyon, D., Junge, A., Wyder, S., Huerta-Cepas, J., Simonovic, M., Doncheva, N. T., Morris, J. H., Bork, P., Jensen, L. J., & Mering, C. von. (2019). STRING v11: protein-protein association networks with increased coverage, supporting functional discovery in genome-wide experimental datasets. *Nucleic Acids Research*, *47*(D1), D607–D613. https://doi.org/10.1093/nar/gky1131

Wilkins, A. D., Venner, E., Marciano, D. C., Erdin, S., Atri, B., Lua, R. C., & Lichtarge, O. (2013). Accounting for epistatic interactions improves the functional analysis of protein structures. *Bioinformatics (Oxford, England)*, *29*(21), 2714–2721. https://doi.org/10.1093/bioinformatics/btt489

Yao, H., Mihalek, I., & Lichtarge, O. (2006). Rank information: a structure-independent measure of evolutionary trace quality that improves identification of protein functional sites. *Proteins*, *65*(1), 111–123. https://doi.org/10.1002/prot.21101
